# From historical dosing to precision dosing: Modernising tobramycin monitoring in people with cystic fibrosis

**DOI:** 10.1007/s00228-026-04205-5

**Published:** 2026-10-17

**Authors:** Ellie Johnson, Michael Williams, Daniel Henderson, Timothy Riddles, David Reid, Daniel Smith, Andrew Burke, Ieuan Edward Shepherd Evans

**Affiliations:** 1 Adult Cystic Fibrosis Centre, The Prince Charles Hospital, Brisbane, QLD Australia; 2 Pharmacy Department, The Prince Charles Hospital, Brisbane, QLD Australia; 3 Faculty of Health, Medicine and Behavioural Science, The University of Queensland, Brisbane, QLD Australia; 4 Lung Inflammation & Infection, QIMR Berghofer Medical Research Institute, Brisbane, QLD Australia; 5 Department of Infectious Diseases, The Prince Charles Hospital, Brisbane, QLD Australia; 6 Department of Thoracic Medicine, The Prince Charles Hospital, Brisbane, QLD Australia

**Keywords:** Cystic fibrosis, Tobramycin, Therapeutic drug monitoring (TDM), Bayesian dosing, Precision dosing, Pharmacokinetics, Aminoglycosides

## Abstract

**Background:**

Historical therapeutic drug monitoring (TDM) strategies for intravenous tobramycin in people with cystic fibrosis (pwCF) were developed in the pre-CFTR modulator era and relied predominantly on clearance-based monitoring to minimise toxicity. Contemporary pwCF demonstrate greater variability in body composition and clinical phenotype, potentially limiting the applicability of historical dosing approaches.

**Methods:**

In December 2024, our adult cystic fibrosis centre implemented Bayesian-guided TDM (TDMx^®^) for intravenous tobramycin. Two serum concentrations obtained during the first dosing interval were used to estimate maximum concentration (C_*max*_), minimum concentration (C_*min*_), and 24-hour area under the concentration-time curve (AUC_0− 24_), enabling model-informed dose individualisation. Outcomes were evaluated in 46 pwCF receiving intravenous tobramycin for pulmonary exacerbations.

**Results:**

Prior to implementation, 74% of pwCF had not undergone formal dose review for more than three years. Following introduction of Bayesian monitoring, 98% received 24-hourly dosing compared with 33% under the historical protocol. Among pwCF with previous admissions, 72% required dose adjustment. Target C_*max*_ (16–20 mg/L) was achieved in 65.2% of participants, while only 17.4% achieved the target AUC_0− 24_ (> 70 mg·h/L); no participant exceeded the upper AUC target of 100 mg·h/L. Thirteen pwCF who would previously have undergone dose reduction because of levels of >1 mg/L at 12 h demonstrated appropriate exposure on Bayesian assessment (mean C_*max*_ 17 mg/L; mean AUC 74 mg·h/L). No nephrotoxicity attributable to the protocol was observed.

**Conclusions:**

Bayesian-guided TDM was feasible, safe, and substantially altered tobramycin prescribing practice. It identified pwCF at risk of both inappropriate dose reduction and underexposure, supporting precision dosing in the evolving CF population.

## Short communication

Intravenous tobramycin, administered in combination with a beta-lactam antibiotic, remains a cornerstone of treatment for pulmonary exacerbations caused by *Pseudomonas aeruginosa* in people with cystic fibrosis (pwCF). However, the therapeutic landscape of CF has changed dramatically over the past decade following the widespread introduction of cystic fibrosis transmembrane conductance regulator modulators (CFTRm), particularly elexacaftor/tezacaftor/ivacaftor (ETI, Trikafta/Kaftrio, Vertex Pharmaceutical^®^) and, more recently, vanzacaftor/tezacaftor/deutivacaftor (VTD, Alyftrek, Vertex Pharmaceutical^®^). CFTRm have improved the nutritional status, including increased body weight, and altered the clinical phenotype of many pwCF, raising important questions regarding the continued applicability of historical antibiotic dosing strategies developed in a pre-modulator era [1–3].

Traditionally, therapeutic drug monitoring (TDM) for tobramycin in CF relied upon clearance-only drug monitoring via single blood sampling, primarily to minimise toxicity [4]. While effective at identifying a risk of toxicity, this approach provides limited information regarding total drug exposure and offers little opportunity for dose optimisation when concentrations are subtherapeutic. At The Prince Charles Hospital Adult Cystic Fibrosis Centre (TPCH ACFC), tobramycin dosing was frequently based on historical regimens used during previous admissions, many of which had not been reviewed for several years. Audit data demonstrated that 34 of 46 pwCF (74%) had not undergone a formal dose review in more than three years, with an average interval between reviews of five years and a maximum of twelve years. This period coincided with widespread uptake of CFTRm therapy often associated with substantial changes in weight and overall health status among the cohort.

Recognising these changes, we implemented a revised intravenous tobramycin protocol in December 2024. The protocol replaced clearance-only drug monitoring with Bayesian-guided TDM using TDMx^®^, a validated pharmacokinetic modelling platform, including in pwCF [5–7]. Two serum concentrations were obtained during the first dosing interval: a peak concentration measured one hour after completion of intravenous infusion and a second sample collected between 6 and 14 h later. These values were used to estimate maximum concentration (C_*max*_), minimum concentration (C_*min*_), and area under the concentration-time curve over 24 h (AUC_0− 24_), allowing individualised dose adjustment based on a more comprehensive assessment of drug exposure. Local pharmacokinetic (PK) targets were derived from established aminoglycoside PK principles, specifically to target C_*max*_:MIC ratio 8–10. Assuming a *P. aeruginosa* MIC of < 2 mg/L, this corresponded to a target C_*max*_ of 16–20 mg/L, C_*min*_ below 0.5 mg/L, and AUC_0− 24_ between 70 and 100 mg·h/L [8]. For MIC > 2 mg/L higher C_*max*_ targets are likely to be required and should be evaluated on an individual basis [9].

We evaluated the impact of this protocol for all pwCF admitted to TPCH ACFC between 1st December 2024 and 30th June 2025. In total, 46 pwCF required admission during this timeframe receiving intravenous tobramycin. The cohort reflected the increasing heterogeneity of contemporary CF populations. Median age was 32.5 years (range 18–64 years). Seventeen participants (37%) were female. Average height was 1.7 m, median weight was 62.5 kg (interquartile range 18.75 kg), and mean body mass index (BMI) was 22.5 kg/m² (SD 4.7). BMI ranged from 13.9 to 38.0 kg/m², demonstrating considerable variation in body composition and reinforcing the limitations of using standardised historical dosing strategies across an increasingly diverse cohort. CFTRm use in our cohort at the time of tobramycin administration was as follows: 32/46 (69.6%) pwCF receiving ETI; 3/46 (6.5%) receiving ivacaftor (Kalydeco, Vertex Pharmaceutical^®^); 1/46 (2.2%) receiving VTD; and 10/46 (21.7%) not receiving CFTRm therapy.

Implementation of Bayesian monitoring resulted in a substantial shift in prescribing practice. Under the previous protocol, only 15 of 46 pwCF (33%) received 24 hourly dosing based on parameters using dosing of 5 mg/kg (based on ideal body weight). Following implementation, 45 of 46 pwCF (98%) were managed with a 24-hour dosing interval, Table 1. This transition reflects increasing confidence in reduced interval aminoglycoside administration when supported by detailed PK modelling and contemporary monitoring methods. More importantly, it facilitated standardisation of practice while maintaining dose individualisation according to individual-specific PK characteristics. There was no clear evidence of a correlation between CFTRm use and the direction of dose adjustment with 5/8 (62.5%) of pwCF not on CFTRm and 13/31 (41.9%) on CTFRm experiencing a tobramycin dose increase with the mean dose changes for each group being + 0.03 mg/kg and −0.09 mg/kg respectively.

**Table 1:** Dosing adjustments at individual levels pre- and post- the introduction of Bayesian modelling TDM for intravenous tobramycin using the TDMx^®^ software. Of the 46 pwCF evaluated seven had not previously received intravenous tobramycin at TPCH ACFC. For the remaining 39 pwCF 28 (71.8%) underwent a dose adjustment, either increased or reduced, following adjusted TDM implementation

Patient number	Historical tobramycin dose (mg)	Historical tobramycin dose (mg/kg)	Historical tobramycin frequency (hourly)	New tobramycin dose (mg)	New tobramycin dose (mg/kg)	New Tobramycin Frequency (hourly)
1	260	2.6	36	320	3.8	24
2	280	6	36	240	4.4	24
3	280	4.4	36	280	4.5	24
4	360	6.1	36	300	4.8	24
5	*No previous admission*			440	5.1	24
6	280	4.9	24	250	5.3	24
7	240	4.1	24	320	5.1	24
8	340	5	36	320	4.8	24
9	320	3	36	360	3.5	24
10	400	6	24	340	4.7	24
11	280	5	36	280	5.2	24
12	200	2.8	24	340	4.9	24
13	*No previous admission*			300	5.5	24
14	320	3.8	24	360	4	24
15	260	4.9	24	260	4.8	24
16	240	5.6	24	240	6	24
17	*No previous admission*			280	4.9	24
18	360	3.9	36	360	3.9	24
19	180	4.7	36	240	5.6	24
20	360	5.8	24	320	4.9	24
21	320	3.6	24	380	4.2	24
22	340	5.9	48	280	5.1	24
23	360	6.7	48	240	4.4	24
24	*No previous admission*			240	5.5	24
25	*No previous admission*			260	5	24
26	360	3	36	480	3.9	24
27	240	4.6	24	260	5.2	24
28	320	5.4	36	240	4.1	24
29	320	4.5	36	360	5.2	36
30	300	5.1	24	280	4.8	24
31	320	4.6	36	320	4.8	24
32	280	3.9	36	360	4.9	24
33	*No previous admission*			320	4	24
34	360	5.3	24	360	5.1	24
35	340	4.7	36	320	4.5	24
36	360	4.9	36	360	4.3	24
37	480	4	24	480	4	24
38	360	5.3	36	340	4.9	24
39	280	5	36	280	4.9	24
39	*No previous admission*			240	5.5	24
39	240	5.2	24	240	5	24
39	360	4.9	36	320	4.1	24
39	320	5.2	48	280	4.7	24
39	300	4.9	48	280	4.8	24
39	240	5.3	36	200	4.3	24
39	240	3.8	24	280	4.2	24

Thirty pwCF (65.2%) achieved the target C_*max*_ of 16–20 mg/L. In contrast, only eight pwCF (17.4%) achieved the recommended AUC target of greater than 70 mg·h/L, although notably no pwCF exceeded the upper limit of 100 mg·h/L. Of the 16 pwCF not achieving the target C_*max*_, seven reached 15 mg/L, five 15.5 mg/L, one 15.7 mg/L and one 21 mg/L. Those pwCF with C_*max*_ levels between 15 and 15.7 mg/L were not adjusted further owing to observed clinical improvement. Two pwCF had levels of 13.5 mg/L and 14.5 mg/L respectively, both these pwCF had limited testing owing to early transition to oral antibiotic therapy and discharge for mild exacerbations. The discrepancy between C_*max*_ and AUC target attainment raises important questions regarding which PK parameter best predicts clinical efficacy in contemporary CF populations. While peak concentration has traditionally been considered the primary determinant of aminoglycoside antibacterial activity, total drug exposure may provide a more comprehensive assessment of treatment adequacy [8, 10, 11]. Evaluating the historical and Bayesian modelling further, we conducted comparative analyses evaluating tobramycin dosing using a paired *t*-test, while dosing frequency was compared using the Wilcoxon signed-rank test due to the frequency being an ordinal discrete variable. PwCF without a previous admission were excluded from the paired analyses. There was no significant difference in dose (mg) (*p* = 0.834), or in weight adjusted dosing (mg/kg) (*p* = 0.607), whereas dosing frequency changed significantly following Bayesian modelling implementation (*p* < 0.001). However, when overall dosing intensity was incorporated into the analysis, there was a mean increase from 238 to 306 mg/day, representing an approximate increase of 29-35.5% in total exposure to tobramycin (*p* < 0.001). (statistical analysis was preformed using GraphPad Prism version 11.1.0 (218)).

The value of Bayesian monitoring becomes particularly apparent when one compares the results with the previous clearance-based approach. Under that historical protocol, 13 pwCF would have warranted a dose reduction due to serum levels > 1 mg/L at 12 h following administration. However, Bayesian analysis revealed that this subgroup had an average AUC of 74 mg·h/L and a mean C_*max*_ of 17 mg/L, indicating appropriate therapeutic exposure. In these pwCF, clearance-based monitoring in the previous protocol may have led to unnecessary dose reduction and an increased risk of under-treatment. This finding highlights a major limitation of relying on isolated concentrations without consideration of the complete PK profile provided by Bayesian modelling and highlights that clearance concentrations alone are not predictive of overall exposure. It is additionally worth considering that historically trough and peak levels do not correlate well with side-effect generation, specifically ototoxicity [12].

Furthermore, two pwCF within this subgroup demonstrated both low peak concentrations (< 15 mg/L) and low AUC values (< 70 mg·h/L). Traditional monitoring would have suggested dose reduction on the basis of elevated serum concentrations, whereas Bayesian modelling indicated that these pwCF may in fact have required dose escalation. Both individuals were tall males with BMIs between 23 and 24 kg/m² and estimated glomerular filtration rates of approximately 70 mL/min/1.73 m², illustrating how conventional monitoring may fail to identify pwCF at risk of subtherapeutic exposure. This observation provides an example of the advantages of model-informed precision dosing and highlights the potential for Bayesian approaches to both prevent toxicity and improve exposure consistent with defined PK targets.

Safety outcomes were reassuring. Thirty-six pwCF (78%) had estimated glomerular filtration rates (eGFR) greater than 90 mL/min/1.73 m², indicating preserved renal function. Despite higher cumulative dosing in many pwCF, repeated blood test surveillance during the admissions showed no features of nephrotoxicity attributable to the protocol implementation within our cohort as indicated by stable eGFR values. No ototoxicity signal was experienced during the timeframe evaluated with baseline audiometry testing performed as routine practice for all pwCF requiring high cumulative dosing of tobramycin. These findings suggest that optimisation of tobramycin exposure through Bayesian-guided monitoring can be achieved safely when supported by structured surveillance and appropriate clinical oversight.

There was no impact on hospital length of stay related to the adjustment in tobramycin TDM with the average length of stay prior to Bayesian-guided TDM being 11.8 days compared to 11.5 days following its implementation. Though this may be due to the fact that most admissions requiring IV antibiotic treatment of exacerbations of CF last between 10 and 14 days. All pwCF managed with adjusted dosing recorded self-reported improvement in symptoms prior to discharge.

The findings also have implications beyond aminoglycoside monitoring alone. Historically, pwCF were characterised by low body weight, chronic illness burden, and relatively homogeneous physiological characteristics. The introduction of CFTRm therapy has fundamentally altered this paradigm [1–3]. Contemporary pwCF increasingly exhibit normal or near-normal nutritional status, wider variation in body habitus, and improved end-organ function. Consequently, assumptions underpinning historical dosing algorithms may no longer be universally applicable, particularly in relation to empirical dosing targeting C_*max*_ levels of greater than 25 mg/L. The substantial inter-individual variability observed within our cohort, combined with the prolonged interval since dose review for many pwCF, underscores the importance of regularly reassessing antimicrobial dosing strategies in the modern CF era.

This study has limitations. It represents a single-centre implementation program involving a relatively small number of pwCF evaluated. Clinical outcomes including microbiological response, and lung function recovery were not assessed. In addition, although the discrepancy between C_*max*_ and AUC attainment is intriguing, the optimal pharmacokinetic target for intravenous tobramycin in CF remains uncertain. Furthermore, this study was not intended to compare alternative empirical dosing regimens, for example those above 5 mg/kg, but rather to evaluate the impact of model-informed dose individualisation on achieved drug exposure. Future multicentre studies correlating pharmacokinetic parameters with clinical outcomes will be necessary to determine whether peak concentration, total exposure, or a combination of both best predicts treatment success in the post-modulator era.

In conclusion, implementation of Bayesian-guided therapeutic drug monitoring for intravenous tobramycin in pwCF was feasible and resulted in substantial changes to prescribing practice with no adverse events. Embedding specialist CF pharmacists to guide these transitions with aminoglycoside dosing helped optimise the approach identifying pwCF who would have been unnecessarily dose-reduced under historical monitoring protocols and revealed instances where dose escalation was required. Bayesian monitoring enabled greater individualisation of therapy, supported widespread adoption of 24-hourly dosing, and achieved therapeutic exposure without evidence of increased nephrotoxicity. As the CF population continues to evolve following the introduction of CFTRm, longstanding antibiotic dosing practices should be regularly re-evaluated to ensure that treatment strategies remain aligned with contemporary individual characteristics and PK requirements. However, it is also worth considering that Bayesian modelling software may be strengthened further by incorporating data from contemporary CF populations on CFTRm therapy. 

## Data Availability

Owing to the clinical nature of the cases, no data will be made available to share.
